# Late initiation of antenatal care and associated factors among pregnant women attending antenatal clinic of Ilu Ababor Zone, southwest Ethiopia: A cross-sectional study

**DOI:** 10.1371/journal.pone.0246230

**Published:** 2021-01-29

**Authors:** Waqgari Tola, Efrem Negash, Tesfaye Sileshi, Negash Wakgari

**Affiliations:** 1 Oromia, TVET Commission, Mettu Health Science College, Mettu, Ethiopia; 2 Faculty of Public Health and Medical Sciences, Mettu University, Mettu, Ethiopia; 3 Department of Midwifery, College of Medicine and Health Sciences, Ambo University, Ambo, Ethiopia; University of South Florida, UNITED STATES

## Abstract

Timely entries to antenatal care have various benefits for pregnant women and birth outcomes. The aim of antenatal care is to assure that every pregnancy culminates in the delivery of a healthy baby without negative effects on the health of pregnant women through health promotion and disease prevention, early detection, and treatment of complications and existing diseases. Hence, this study assessed the late initiation of antenatal care and associated factors among pregnant women attending antenatal clinics at public health centers of Ilu Ababor Zone, southwest Ethiopia. An Institution-based cross-sectional study was carried out among 389 pregnant women who were attending antenatal care service at twelve randomly selected health centers. A systematic sampling technique was employed to recruit pregnant women. Pretested and structured questionnaires were used to collect data. Data were entered into Epidata and exported to SPSS for analysis. Those women who started antenatal care follow up after 12 weeks of gestational age were categorized as booked lately. Bivariable and multivariable logistic regression was employed to identify an association between the independent predictors and the outcome variable. In this study, 277 (71.2%) of the participants were booked their first antenatal care visit lately. Having family size of ≥ 4 (AOR: 2.25; 95% CI: 1.07–4.74), maternal age ≥ 25 years (AOR: 2.30; 95%CI: 1.02–5.18) and perceived the right time of booking > 12 weeks of gestation (AOR: 2.39; 95% CI: 1.13–5.04) had higher odds of late antenatal care initiation. Similarly, not being a member of women’s health developmental army (AOR: 2.35; 95%CI: 1.09–5.07) and ANC not attended previously (AOR: 3.32; 95% CI: 1.17–9.42) had also a more likelihood of booking antenatal care lately. In this study, the majority of women started antenatal care lately. Thus, the provision of health education on the importance of attending first antenatal care early is recommended.

## Introduction

The care provided by skilled health care professionals to pregnant women and adolescent girls in order to ensure the best health conditions for both mother and baby during pregnancy is considered Antenatal care (ANC) [1]. The World Health Organization (WHO) previously recommends a minimum of four ANC visits: ideally, the first visit should occur before 16 weeks of gestation [2, 3]. However, recently WHO recommends a minimum of eight contacts: with the first contact scheduled to take place in the first trimester (up to 12 weeks of gestation) [4]. This new recommendation reduces maternal and perinatal deaths by increasing the opportunity of maternal and fetal assessment to notice complications and get better communication between providers and mothers [5]. Vitamin D supplementation, screening for infections, down’s syndrome, and hematological conditions such as sickle cell diseases and thalassaemias are some of the services which are provided during this period [1]. By supplying iron and folic acid during the first trimester of pregnancy the risk of anencephaly and spinal bifida can be minimized [4]. Inadequate care during this time breaks a critical link in the continuum of care and affects both women and babies [3, 6]. In spite of this, many women in sub-Saharan Africa tend to wait to start antenatal care until the second or third trimester of pregnancy [7].

Globally, approximately 830 women died every day due to complications during pregnancy and childbirth in 2015 [8]. About 99% of maternal deaths and stillbirths occur in low-resource settings and of which most of them can be prevented through early ANC service provision [9–12]. Thus, ANC remains one of the means to reduce maternal deaths which can be provided at the lower level health facilities [13, 14]. Early ANC visits can lower maternal deaths by identifying complications early and giving an opportunity to be screened for HIV timely and receive testing and treatment for syphilis [15].

Health and access to basic health services are fundamental human rights and prerequisites for economic growth and social inclusion [3]. Though good progress has been made in the total number of ANC visits, the prevalence of late ANC initiation is still high in developing countries including Ethiopia [16]. Failure to attend antenatal care at an early period can result in potential complications during pregnancy, delivery, and puerperium [17]. Moreover, previous studies have identified factors for late booking of ANC such as maternal education, husband education, age, parity, type of pregnancy, unemployment, lack of knowledge or misconceptions about the purpose of antenatal care, marital status, socioeconomic status, financial constraints, and problems in the last delivery or pregnancy [17, 18]. However, the studies reporting about late initiation of ANC and associated factors are limited in the study settings, considering the variation of the socio-economic characteristic of the study population which may influence the time of ANC initiation. Hence, the result of this study helps health policymakers and other stakeholders to develop public health policies and enhance family and social support system for pregnant women in the communities. As a result, the purpose of this study was to assess the magnitude of late initiation of antenatal care and associated factors among pregnant women attending antenatal clinics at public health centers of Ilu Ababor Zone, southwest Ethiopia.

## Methods and materials

### Study design, setting, and population

An institution-based cross-sectional study design was conducted in Ilu Ababor zone, Oromia regional state, southwest Ethiopia from June to July 2018. Ilu Ababor zone is located 575 kilometers from Addis Ababa, the capital city of Ethiopia. The zone has 14 administrative districts. Regarding health facility, there are 2 hospitals, 39 health centers, and 273 health posts in the zone. All pregnant women attending ANC in public health centers of Ilu Ababor zone during the study period were considered as the source population whereas all pregnant women who attending ANC service in the selected public health centers were considered as the study population.

#### Sample size determination and sampling procedure

The sample size was calculated using a single population proportion formula n = (Z α/2)^2^ P (1-P)/ d^2^ based on the following assumptions: the proportion (P)of late initiation of ANC 64% which was taken from the previous study [19], 95% the confidence level of Z α/2 = 1.96, 5% of absolute precision. Thus, with a non-response rate of 10%, the minimum sample size needed for the current study was 389 pregnant women.

Among 39 health centers in the Ilu Ababor zone, 12 health centers were selected using a simple random sampling technique. An average monthly ANC visit caseload of each selected health center was assessed before proceeding to the study. Then, the sample size was allocated for the study facilities using population proportion to size for each selected health center. Finally, at each health center, the study subjects were interviewed during the initial or follow-up of antenatal service. Accordingly, 1188 clients were received ANC from all studied institutions and the sampling frame was calculated as 1188/389 ≈ 3. Every third client from all health institutions was interviewed by using a systematic sampling technique. The first client to be interviewed was selected randomly. Accordingly, the number of clients in a month for the selected 12 health centers were (Alge = 132, Suphe = 66, Bacho = 88, Gore = 132, Nopa = 88, Burusa = 66, Yayo = 132, Hurumu = 110, Uka = 66, Bure = 132, Kidame = 88 and Lalo = 88). The proportional allocation for twelve health centers depending on their client load is presented as follows:

$$nh=\frac{Nh}{N}\times n$$

◦ n_h_ is the sample size which was allocated to health center h
◦ N_h_ is the number of clients who visited the selected health center h in a month
◦ N the cumulated number of clients who visited all the twelve health centers in a month
◦ n is the total sample size to be allocated and “h’ runs from 1 to 12.

#### Data collection tools and procedures

The data were collected using structured interviewer-administered questionnaires. Different relevant literatures were reviewed to develop a tool that addresses the objectives of the study (2, 14–16, 19–29). The exit interview technique was used to collect data about their socio-demographic characteristics, individual-related factors, decision-making status, and obstetric history of the women. The questionnaire was originally developed in English, and translated into Afan Oromo. Finally, it was translated back to English by a translator to ensure its consistency.

#### Data quality assurance

The quality of the questionnaire was assured by properly designing and pre-testing the tool and training the data collectors and supervisors before the actual data collection. The questionnaires were pre-tested among 19 pregnant women who were attending ANC at Chora health center in the Buno Bedele zone before actual data collection. To verify the internal consistency of the instrument, Cronbach’s alpha analysis of past obstetrics, current pregnancy, ANC visit time, and women’s decision-making status-related questionnaire parts were done with the value of 0.72 which implies that the tool items have high internal consistency. Moreover, two days of training was given to data collectors and supervisors on the data collection techniques and procedures. Completeness and consistency of the collected data were reviewed and checked by supervisors and investigators.

### Operational definition

#### Late initiation of antenatal care

If a woman came for ANC beyond 12 weeks of gestation for the first time during the pregnancy; she was considered as having late initiation of antenatal care [late booking], otherwise considered as early booking visit (within the recommended time) [4].

#### Women’s health development army

Enormous voluntary structural arrangement that involves women’s development team and one-to-five connections to advance population health and renew the country [20].

### Data analysis procedures

The collected data were cleaned, edited, coded, and entered into Epidata version 4.1 and exported to SPSS version 20.0 for analysis. The collected data were checked for inconsistencies and missing values. After categorizing and defining variables, descriptive analysis was carried out for each of the independent variables. Multicollinearity among the independent variables was assessed by using variance inflation factors. However, no significant multicollinearity was detected as the variance inflation factor for all variables was less than five. Both bivariable and multivariable logistic regression analyses were used to determine the association of each independent variable with the outcome variable. On bivariate analysis variables that showed significant association at a p-value of< 0.25 were entered to multivariable analysis to select factors associated with the late initiation of ANC. The screened variables were fitted to the multivariable logistic regression model through a backward stepwise method to reduce the effects of cofounders. Hosmer and Lemeshow goodness of fit test was used to assess how the data fits the model *(*P = 0.785*)*. Crude (COR) and adjusted (AOR) odds ratios with their 95% confidence intervals (CIs) were estimated to identify the presence and strength of associations, and statistical significance was declared if p < 0.05. Moreover, a chi-square test was performed to compare booked timely and booked late by the socio-demographic characteristics.

### Ethics statement

The ethical approval letter was obtained from Mettu University ethical review committee. Then a formal letter that explains the objectives, rationale, and expected outcomes of the study was written to the respective health institutions. The respondents were informed about the objective and purpose of the study. Finally, written informed consent was obtained from each study subject or from their parent or guardian for participants under 18years prior to the data collection process. In addition, the confidentiality and privacy of the study participants were assured and respected.

## Results

### Socio-demographic characteristics of the respondents

Overall, three hundred eighty-nine pregnant women who were attending antenatal care service at public health centers were interviewed. One hundred forty-seven (37.8%) of them were in the age group of 15 to 24 years. The mean ages of respondents were 23.54 years (SD± 4.5), and the age ranges from 15 to 41 years. The majority, 340 (87.4%) of them were Oromo in ethnicity. One hundred fifty (38.6%) of them were Protestant religious followers. About two-thirds, 242 (62.2%) of the respondents had a family size of less than or equal to three (Table 1).

**Table 1:** Socio-demographic characteristics of the respondents, Ilu Ababor Zone, Oromia region, southwest Ethiopia, 2018.

Variables (n = 389)	Number (%)	^α^Booked timely	^αα^Booked lately	Chi-square test (1-sided)
		Number	Number	P-value
**Age**				0.001
<25 years	148 (38.1)	64	84	
≥25 years	241 (61.9)	48	193	
Percent	100	28.8	71.2	
**Marital status**				0.536
Single	13 (3.3)	3	10	
Married	362 (93.1)	106	256	
Other*	14 (3.6)	3	11	
Percent	100	28.8	71.2	
**Residence**				0.005
Urban	147 (37.8)	54	93	
Rural	242 (62.2)	58	184	
Percent	100	28.8	71.2	
**Religion**				0.257
Protestant	150 (38.6)	42	108	
Muslim	120 (30.8)	42	78	
Orthodox	118 (30.3)	28	90	
Others**	10 (0.3)	0	10	
Percent	100	28.8	71.2	
**Ethnicity**				0.442
Oromo	340 (87.4)	101	239	
Amhara	34 (8.7)	6	28	
Tigre	13 (3.3)	4	9	
Others^**‴**^	2 (0.6)	1	1	
Percent	100	28.8	71.2	
**Educational status**				0.000
No formal education	152 (39.1)	26	126	
Primary education	125 (32.1)	42	83	
Secondary school and above	112 (28.8)	44	68	
Percent	100	28.8	71.2	
**Family size**				0.000
≤3	242 (62.2)	87	155	
≥4	147 (37.8)	25	122	
Percent	100	28.8	71.2	
**Husband education**				0.503
No formal education	86 (23.4)	22	64	
Primary school (1–8)	137 (37.2)	38	99	
Secondary school (9–12) and above	145 (39.4)	47	98	
Percent	100	28.8	71.2	
**Distance to a health facility**				0.011
<1 hour	141 (36.2)	51 (45.5)	90 (32.5)	
≥1hour	248 (63.8)	61 (54.5)	187 (67.5)	
Percent	100	28.8	71.2	

^α^ **=** ANC initiated before or at 12 weeks of gestation,

^αα^ **=** ANC initiated after 12 weeks of gestation

* = Divorced, Widowed,

** = Catholic, Waqeffata,

‴ = Gurage, Keffa.

**Timing of first antenatal care visit.** The proportion of respondents who initiated ANC lately were 277 (71.2%) [CI: 68.2–74.6]. The timing of the first ANC booking among respondents ranged from 4 to32 weeks of gestation. The mean gestational age at which the pregnant women booked the first ANC was 17.2 ± 5weeks. Moreover, the timing for the women who attend ANC lately ranged from 13 to 32 weeks of gestation with 23 medians of weeks. Among the total respondents, 161 (41.4%) of them reported that they were informed about when to book for their first ANC. Of those who were not informed and advised on time of first ANC booking 177 (63.9%) of them started their ANC visit lately (Table 2).

**Table 2:** Timing of ANC visit and related characteristics of respondents, Ilu Ababor Zone, Oromia region, Southwest Ethiopia, 2018.

Variables (n = 389)	Number (%)	Booked Timely	Booked lately
		Number (%)	Number (%)
**Heard the existence of ANC**			
Yes	378 (97.2)	109 (97.3)	269 (97.1)
No	11 (2.8)	3 (2.7)	8 (2.9)
Percent	100	28.8	71.2
**Informed when to book to first ANC**			
Yes	161 (41.4)	61 (54.5)	100 (36.1)
No	228 (58.6)	51 (45.5)	177 (63.9)
Percent	100	28.8	71.2
**Member of women health development army**			
Yes	192 (49.4)	65 (58.0)	104 (37.5)
No	197 (50.6)	47 (42.0)	173 (62.5)
Percent	100	28.8	71.2
**Presence of ambulance service**			
Yes	346 (88.9)	108 (96.4)	238 (85.9)
No	43 (11.1)	4 (3.6)	39 (14.1)
Percent	100	28.8	71.2
**Having final say on using current ANC**			
Women alone	44 (11.3)	12 (10.7)	32 (11.6)
Husband	132 (33.9)	23 (20.5)	109 (39.4)
Jointly	213 (54.8)	77 (68.8)	136 (49.1)
Percent	100	28.8	71.2

Regarding the reason for the late initiation of ANC; more than half (51.3%) perceived that it was the right time, while nearly a quarter (23.8%) did not recognize that they were pregnant. In contrast, 6.5% and 14.4% of mothers were inattentive and did not know the right time for ANC booking respectively.

#### Obstetric history of the respondents

One hundred and eighty-eight (48.3%) of the respondents were primigravida. Most 188 (93.5%) of the women had no history of stillbirth (Table 3).

**Table 3:** Obstetric characteristics of the respondents, Ilu Ababor Zone, Oromia region, Southwest Ethiopia, 2018.

Variables (n = 389)	Booked Timely	Booked lately	Total
	Number (%)	Number (%)	Number (%)
**Gravidity**			
Primigravida	62 (55.4)	126 (45.5)	188 (48.3)
Multigravida	50 (44.6)	151 (54.5)	201 (51.7)
**Parity**			
Para zero	63 (56.2)	129 (46.6)	192 (49.4)
Para one and above	49 (43.8)	148 (53.4)	197 (50.6)
**History of stillbirth (N = 201**)			
Yes	4 (8.0)	9 (6.0)	13 (6.5)
No	46 (92.0)	142 (94.0)	188 (93.5)
**Attended ANC for past pregnancy (N = 201)**			
Yes	45 (90.0)	97 (64.2)	142 (70.6)
No	5 (10.0)	54 (35.8)	59 (29.4)
**Had complication in a previous pregnancy (N = 201)**			
Yes	4 (8.0)	14 (9.3)	18 (9.0)
No	46 (92.0)	137 (90.7)	183 (91.0)
**Means of current pregnancy recognition**			
Urine test	56 (50.0)	96 (34.7)	152 (39.1)
Missing period	56 (50.0)	181 (65.3)	237 (60.9)
**Pregnancy planned**			
Yes	100 (89.3)	210 (75.8)	310 (97.7)
No	12 (10.7)	67 (24.2)	79 (20.3)

#### Factors associated with the late first ANC visit

On bivariate analysis, factors found to be significantly associated withthe late booking of first ANC visitwas age, family size, residence, perceived booking time, parity, gravidity, distance to the health facility, pregnancy plan, member of women health development army, previously attended ANC, means of recognizing pregnancy and presence of ambulance service. On multivariable analysis, age, family size, perceived booking time, member of women health development army, and previously attended ANC were significantly associated with the late booking of the first ANC visit.

Women aged 25 years and above had 2.30 (AOR: 2.30; 95% CI: 1.02, 5.18) times higher odds of late first ANC visit as compared to the women aged less than 25 years. Those respondents living within a family size of four and above had 2.25 (AOR: 2.25; 95% CI: 1.07, 4.74) times higher odds of late first ANC visit as compared to those who were living within three and less family size.

A woman who was not a member of the women’s health development army had 2.35 (AOR: 2.35; 95% CI: 1.09, 5.07) times higher odds of late first ANC visit compared to those who were woman health development army members. Respondents who perceived the right time of booking greater than 12 weeks of gestation had2.39 (AOR: 2.39; 95% CI: 1.13, 5.04) times higher odds of late first ANC visit as compared with women who perceived the right time of booking at or before 12 weeks of gestation. Women who had not attended ANC during their previous pregnancy had 3.32 (AOR: 3.32; 95% CI:1.17, 9.42) times higher odds of late first ANC visit as compared to women who attended antenatal care during the past pregnancy (Table 4).

**Table 4:** Multivariable model of factors for late first ANC initiation among pregnant women attending health centers of Ilu Ababor Zone, Oromia region, Southwest Ethiopia, 2018.

Variables	COR [95% CI]	AOR [95% CI]	[AOR]
			P-value
**Age**			
<25 years	1	1	
≥25 years	3.06 (1.97, 4.90)	2.30 (1.02, 5.18)*	0.035
**Residence**			
Urban	1	1	
Rural	1.84 (1.17, 2.87)	1.24 (0.82, 2.52)	0.075
**Distance to the health facility**			
<1hour	1		
≥1hour	1.73 (1.10, 2.72)	1.56 (0.79, 1.69)	0.061
**Family size**			
≤3	1	1	
≥4	2.73 (1.65, 4.53)	2.25 (1.07, 4.74)*	0.003
**Pregnancy plan**			
Planned	1		
Not planned	2.65 (1.37, 5.13)	2.17 (1.0, 5. 67)	0.055
**Gravidity**			
Primigravida	1		
Multigravida	1.48 (0.95, 2.31)	1.19 (0.46, 3.33)	0.079
**Parity**			
Para zero	1		
Para 1 and above	1.47 (0.94, 2.29)	1.02 (0.34, 1.69)	0.085
**Perceived right booking time**			
≤12wks of gestation	1	1	
>12 wks of gestation	3.57 (2.22, 5.75)	**2.39 (1.13, 5.04)** *	0.001
**Member of women health development army**			
Yes	1	1	
No	2.30 (1.47, 3.59)	**2.35 (1.09, 5.07)** *	0.001
**Previous attended ANC**			
Yes	1	1	
No	5.01 (1.87, 13.37)	**3.32 (1.17, 9.42)** *	0.001
**Means of recognizing pregnancy**			
Urine test	1		
Missing period	1.88 (1.20, 2.94)	1.41 (0.83, 2.13)	0.062
**Presence of ambulance service**			
Yes	1		
No	4.42 (1.54, 12.69)	3.32 (0.71, 11.68)	0.081

*Indicates significant association at p-value <0.05, 1 = Reference, COR: Crude odds ratio, AOR: Adjusted odds ratio.

## Discussion

This study assessed the late initiation of first antenatal care and associated factors among pregnant women attending antenatal clinics at public health centers of Ilu Ababor Zone, southwest Ethiopia. About two-thirds, 277 (71.2%) of the respondents had started their first ANC lately. Overall, the magnitude of late first ANC visit in this study is higher compared with the WHO recommendation, which is the first contact should be initiated before 12 weeks of gestation [4] and the studies were done in Malaysia 56.2% [21] and Addis Ababa 59.8% [22]. However, this finding is similar to the studies conducted in Isra University Hospital 71% [23] and Zambia 72% and 68.6% in rural and urban districts respectively [15]. Moreover, the proportion of respondents who booked late in this study is lower than the 2016 Ethiopian demographic health survey report of 80% [24] and Ambo, Ethiopia 86.8% [18]. This could be explained by differences in the year of study and a lot of works has been done since then to improve maternal health services.

In this study, women aged 25 years and above had higher odds of late first ANC visit than women aged less than 25 years. This finding is supported by studies done in Gondar, Ethiopia [25], and East Wollega zone, Ethiopia [26]. The possible explanation for this could be young women may consider themselves at high risk of pregnancy complications and therefore take the needed precautions to avert any unforeseen maternal complications. Another reason could be older women may also feel more confident from previous experience and may feel starting ANC early is unnecessary.

Also, pregnant women with a family size greater than or equal to 4 persons were about two times more likely to initiate the first ANC lately than women with a family size of three and lower. This finding is in line with the studies done in Rwanda [27] and the Hadiya zone, Ethiopia [28]. This could be explained by financial constraints which increase as the family size increases. The other possible explanation might be because women spend more time on their multiple responsibilities for the care of children, and all other household activities than on their own health.

As the time of the first ANC visit varies from one respondent to another, there is also a difference in the perceived right time of the first ANC. This study revealed that women who perceived the right booking time of first ANC beyond 12 weeks of gestation had higher odds of late first ANC visit as compared to those who perceived the right booking time of first ANC at or less than 12 weeks of gestation. This finding is supported by the studies done in different parts of Ethiopia [25, 29, 30]. The possible reason for this could be that individuals seek and practice what they usually know and to the level of their perception.

Respondents who did not attend ANC preceding the current pregnancy were three times more likely to commence ANC after the first trimester of the pregnancy. This finding is in line with the studies done in different parts of Ethiopia [31, 32]. Furthermore, women who were not a member of women’s health development army were about two times more likely to visit antenatal care lately compared to mothers who were members of the women’s health development army. This could be due to government structured networks working closely with health extension workers and strive to create demand for the health not only for women of reproductive age but also for the community as a whole.

### Limitations of the study

Firstly, due to the nature of the study design, this study is subjected to selection and measurement bias and it is difficult to establish temporal relationships. Although the authors used a sampling frame in hopes of creating a more representative sample, selection bias may still be apparent and made the results non-generalizable. Secondly, the exclusion of pregnant women who attended antenatal care at private health facilities which lead to a lack of generalization was considered a limitation of this study. This study could fill various gaps in information regarding the late initiation of antenatal care and associated factors in the study population.

## Conclusions

In this study, the majority of women started their first antenatal care contact lately. In addition, maternal age, family size, ANC not attended previously, being a member of women’s health development army, and perceived right time of booking for ANC were factors identified for late first ANC initiation. Provision of health education on the importance of attending the first ANC visit as per the WHO recommendations and strengthening women’s health development army within the community is recommended.

## Supporting information

S1 File Questionnaire: Afaan Oromo version. (DOCX)

S2 File Questionnaire: English version. (DOCX)

S1 Dataset (SAV)

## Abbreviations

ANC: Antenatal care
WHO: World Health Organization
