# Supplementary material for: Late initiation of antenatal care and associated factors among pregnant women attending antenatal clinic of Ilu Ababor Zone, southwest Ethiopia: A cross-sectional study
Source: PLoS One. 2021 Jan 29;16(1):e0246230. doi: 10.1371/journal.pone.0246230 (PMC7845970; doi:10.1371/journal.pone.0246230)
Supplement: S1 File — (DOCX) [file pone.0246230.s001.docx]

# **QUESTIONNARE: AFAAN OROMO VERSION**

**Guca odeeffannoo fi waliigaltee**

**Mata-duree: -**Qorannoon Kun bara 2010 Godina Ilubabor keesatti tilmaama dubartoota ulfaa kunuunsa dahumsa duraa isa jalqabaa yeroon argachuu hin dandeenye fi sababoota isaan yeroo kaa’ame keessatti argamuu hin dandeenyeef sakatta’uu ilaallata.

**Dhaabbata**: Muummee Fayyaa Hawaasaa, Faakaltii Meedikaalaa fi Saayinsii Fayyaa Yuunversiitii mattuu

**Baasii Qo’annoo kan deggaru**: dhuunfaa

**Kaayyoo qo’annichaa:** Kaayyoon qo’annichaa inni ijoon tilmaama dubartoota ulfaa kunuunsa dahumsa duraa isa jalqabaa yeroon argachuu hin dandeenye fi sababoota isaan yeroo kaa’ame keessatti argamuu hin dandeenyeef sakatta’uu ta’a. Bu’aan qorannoo kanaa haadholiin ulfaa tajaaajila kunuunsa dahumsa dura isa jalqabaa akka isaan yeroon deemanii hin jalqabne waan isaan taasisu baruun akkaataa qajeelfama jiruun akka itti fayyadaman offisaaniif daa’ima isaanii fayya qabeessa tahe argachuuf isaan gargaara.

**Haala Addeemsa Qorannichaa:**Qo’annoo kun gaafile qophaa’aniin gargaramudhaan tilmaama dubartoota ulfaa kunuunsa dahumsa duraa isa jalqabaa yeroon argachuu hin dandeenye fi sababoota isaan yeroo kaa’ame keessatti argamuu hin dandeenyeef sakatta’uu fi madaaluun adda kan baasnu ta’a. Eeyamin koree namussa Yuunversiitii mattuu itti ansuun qajeelcha egumsa fayyaa gdina iluu abbaa boor irraa ni fudhatama. Saan booda itti gafataamaa buufataalee fayyaa wal quunamun haali ni mijeeffama. Dhumaratii namoonni qo’annoo irratti gaggeefamu irra waliigalteen afaanii godhamee odeeffannoon ni guurama.

**Miidhaa-qo’annoo:** kana irratti hirmaachuun yeroo keessaan hanga daqiiqaa 25 kan hin caalle isin jalaa fudhachuu danda’a malee miidhaa addaa isin irraan ga’u hin qabu.

**Bu’aa:** Qorannoo kana keessatti hirmaachuun bu’a kallattiin isin argattan jiraachuu baatullee hirmaachuun keessaaniin isin dabalatee haadholiin ulfa godina kana keessa jiran sababoota yeroon tajaajila kunuunsa duraa isa jalqabaaa yeroon hin eegalleef adda bahe irratti hundaahuun kallattii furmaataa xiinxaluuf ni gargaara.

**Kaffaltii hirmaannaa:** kanfaltiin mallaqaan ykn faayidaan addaa waan qo’annoo kana irratti hirmaatteef siif kennamu hin jiru.

**Iccitii:** Maqaan kee gaffii irratti hin barreeffamu. Deebiin ati kennitu hundi haala amansiissaadhaan qabama.Ega odeeffannoon sassaabamee boodaa waraqaan gaaffii ati irratti guutte sanduqa keessatti naqamee cufamee taa’a. maqaan kee waan irra hin jirreef lakkoofsa addaa keennameen eegamee qoratticha fi gargaartotaaan qofa illaalama.

**Qorannicha irratti hiramaachuu ilaalchisee**: Qorannoo kanarratti hirmaachuu ilaalchisee guutumaan guutuutti eeyyama kee irratti hundaa’a. Yeroo barbaade addaan kute bahuuf mirga ni qabda kanaafis miidhaan sirra ga’u gonkumaa hin jiru.

**Namoota argachuu dandeessan:** Projeekitiin qo’annoo kanaa koree namussa Yuunversiitii mattuun gulaalmee mirkanaa’ee jira. waa’ee qo’annaa kanaa ilaalchisee odeeffannoo dabalaata yoo barbadaan koree hasoofisisuun ni danda’ama. Qo’annoo kana ilaalchisee gaaffii yoo qabaattan namoota armaan gadii hasofsiisuu dandeessu.

**Qo’annoo kana gaggeessan:** Waaqgaari TolaaMobile**:** 091-09-33-30

**Gorsittoonni Qo’annichaa: 1**. Efrem Negaash (MSc/Nutrition)Mobile**:** 0911-53-59-29

**2.** Tesfaayee Sileshii (MPH/Epidemiology)Mobile**:** 0911-81-06-42

**Maqaa Buufata Fayyaa**__________________

**Kutaa 1: Gaaffilee hawwaasummaan(hawaas-diinagdeen) walqabatan**

| Lakk | Gaaffii | Koodii haadha ulfaa--------------- | Yaada |
| --- | --- | --- | --- |
| 1 | Umuriin kee meeqa? | waggaa--------------- |  |
| 2 | Sadarkaa gaa’elaa | 01. Hin Heerumne  02. Heerumeera  03. Wal Hiikeera  04. Kan Abbaan Manaa irraa Du’e  05.A/Manaa waliin iddoo garaa garaa jiraatu | Yoo hin heerumne tahe Lakk.gaaffii 11 fi 12 irra utaali |
| 3 | Bakka jiraatan(dhufan) | 01. Magaalaa  02. Baadiyyaa |  |
| 4 | Amantaan keessan maal? | 01.Prooteestaantii  02.Musliima  03.Ortodoksii  97.Kan Biraa(Ibsi)---- |  |
| 5 | Sabummaan kee maal? | 01. Oromoo  02. Amaara  03. Tigiree  04. Guraagee  97. Kan biraa (ibsi)--- |  |
| 6 | Hojiin kee maal? | 01. Haadha Warraa  02. Qonnaan Bulaa  03. Dafqaan Bulaa  04. Daldalaa  05. Hojjetaa Mootummaa  06. Hojii dhuunfaa  07. Barataa  97. Kan Biraa (Ibsi)--- |  |
| 7 | Sadarkaan barumsaa kee meeqa? | 01.Barreessuu fi dubbisuu kan hin dandeenye  02.Barreessuu fi dubbisuu kan dandeessu  03. Sadarkaa 1^ffaa^ (1-8)  04. Sadarkaa 2^ffaa^ (9-12)  05.Kolleejjii fi isaa oli |  |
| 8 | Galiin maatii kee hangam ta’a ji’atti? | ---------- Qr. |  |
| 9 | Manni jireenyaa kee B/ fayyaa kana irraa hangam fagaata? | sa’atii ----------- |  |
| 10 | Baayinni Maatii kee meeqa? | ---------------------- |  |

| 11 | Hojiin Abbaa manaa kee maal? | 01. Qotee Bulaa  02. Dafqaan Bulaa  03. Daldalaa  04. Hojjetaa Mootummaa  05. Hojii dhuunfaa  06. Barataa  97. Kan Biraa (Ibsi)--- |  |
| --- | --- | --- | --- |
| 12 | Sadarkaa barumsaa Abbaa manaa kee meeqa? | 01.Barreessuu fi dubbisuu hin danda’u  02.Barreessuu fi dubbisuu ni danda’a  03. Sadarkaa 1^ffaa^ (1-8)  04. Sadarkaa 2^ffaa^ (9-12)  05.Kolleejjii fi isaa oli |  |

**Kutaa 2: Gaaffilee ulfaa fi yeroo dahumsaan wal qabatan**

| 13 | Ulfi kee kun isa jalqabaa keeti? | 01. Eeyyee  02. Lakkii | Yoo eeyyee tahe gara lakk.20 deemi |
| --- | --- | --- | --- |
| 14 | Hanga yoonaatti si’a meeqaaf ulfa taatee beekta? | __________ |  |
| 15 | Hanga yoonaatti si’a meeqa deesse jirta? | __________ |  |
| 16 | Rakkoon mucaan garaatti du’ee dhalatu si mudate beekaa? | 01. Eeyyee  02. Lakkii |  |
| 17 | Kanaan dura ulfi si irraa bahee/of irraa baastee beekaa? | 01. Eeyyee  02. Lakkii |  |
| 18 | Ulfa kee kanaan duraatiif tajaajila kunuunsa dahumsa duraa hordoftee beektaa? | 01. Eeyyee  02. Lakkii |  |
| 19 | Ulfa kee kanaan duraatiif mallattoowwan balaa cimaa yeroo ulfaa, ciniinsuu fi dahumsaa si mudatee beekaa? | 01. Eeyyee  02. Lakkii |  |
| 20 | Amma ulfa tahuu kee kana jalqaba maaliin adda baaste? | 01. Xurii/ laguun koo waan hafeef  02. Fincaan koo qorachiisuutiin  97. Kan biraatiin (ibsi)-------- |  |
| 21 | Ulfi kee ammaa kun abbaa manaa fi maatii kee biratti fudhatama qabaa? | 01. Eeyyee  02. Lakkii |  |
| 22 | Ulfi kee ammaa kun dursamee karoorfameeraa? | 01. Eeyyee  02. Lakkii |  |

**Kutaa 3: Gaaffilee yeroo hordoffii KDD fi nama dhuunfaan walqabatan**

| 23 | Tajaajila kunuunsa dahumsa duraa isa jalqabaa Ulfa ji’a meeqaa taatee eegalte? | | Ji’a----------- |  |
| --- | --- | --- | --- | --- |
| 24 | Maaliif yeroo sanatti/kanatti ilaalamuu deemtee? | | 01.Yeroo sirrii waan natti fakkaateef  02.Yeroo isa sirrii tahee fi faayidaa isaa irratti hubannaa gahaa waantan hin qabneef  03.Ulfa tahuu koo waan hin beekneef  04. Hin barbaachisu jedhee waantan yaadeeef  05. Kan biraa(ibsi)-------- |  |
|  |  | |  |  |
| 25 | Tajaajilli kunuunsa dahumsa duraa jiraachuu isaa dhageessee beekta? | | 01. Eeyyee  02. Lakkii |  |
| 26 | Haati ulfaa tokko kunuunsa dahumsaa isa duraa yoom akka eegaluu qabdu dhageessee beekta? | | 01. Eeyyee  02. Lakkii |  |
| 27 | Tajaajila kunuunsa dahumsa duraa isa jalqabaa haati ulfaa tokko argachuu kan qabdu yoom jettee yaada? | | Ji’a-------------- |  |
| 28 | Yoo dhageessee beekta tahe maddi odeeffannoo kee eenyu ture? | | 01. Ogeessa Fayyaa  02. Firoota ykn Hiriyoota koo irraa  03. Miidiyaa (TV,Raadiyoo ) irraa  04. kan biroo (ibsi)------------------ |  |
| 29 | Miseensa ijaarama RMDD (1-5) keesssa jirtaa? | | 01. Eeyyee  02. Lakkii |  |
| 30 | Tajaajilli Ambuulaansii naaannoo kee jiraa? | | 01. Eeyyee  02. Lakkii |  |
| 31 | Maatii waliin jiraattu keessaa hojiilee kanneeniif eenyutu murtee dhumaa kennaa? | 30.1.Waayee fayyaa dhuunfaa keetii ilaalchisee | 01. Qofaa koon murteessa  02. Abbaa Manaa kootu murteessa  03. waliin murteessina  04. kan biraa(ibsi)--------- |  |
|  |  | 30.2.Waayee amma Kunuunsa Dahumsa Duraa fayyadamuu kee ilaalchisee | 01. Qofaa koon murteessa  02. Abbaa Manaa kootu murteessa  03. Waliin Murteessina  04. Kan biraa(ibsi)--------- |  |

**Gumaacha Keessaniif Galatoomaa!**
