# Supplementary material for: Late initiation of antenatal care and associated factors among pregnant women attending antenatal clinic of Ilu Ababor Zone, southwest Ethiopia: A cross-sectional study
Source: PLoS One. 2021 Jan 29;16(1):e0246230. doi: 10.1371/journal.pone.0246230 (PMC7845970; doi:10.1371/journal.pone.0246230)
Supplement: S2 File — (DOCX) [file pone.0246230.s002.docx]

# **QUESTIONNARIE: ENGLISH VERSION**

Questionnaires to assess factors associated with late antenatal care booking among pregnant mothers attending ANC clinics at selected public health facilities in Ilu ababor Zone, south West Ethiopia. Hello, my name is _______________________and working with Waqgari Tola and I am a member of this research team. As part of this survey, we are collecting information on proportion and factors associated with late initiation of Antenatal care among pregnant women attending Antenatal clinic at selected public health facilities in Ilu ababor Zone, South West Ethiopia. We will include all mothers who came for usual ANC visit. The questionnaire includes socio-demographic, individual related factors and obstetric factors. I would like to assure you that the study is confidential. We will not keep a record of your name and address. Only honest answers would contribute for improvement of health planning. Therefore, you are kindly requested to respond genuinely and voluntary with patience. The survey will take about 25 minutes. Would this be okay for you? I understood about the advantage of the research and the roles I will have in the research.

I have agreed to participate in the research. A. Yes B. No
If respondent agrees to be interviewed,
Starting time ___________________: End time: _________________
Thank you for your participation!
Date of data collection _________________________________________________
Name of data collector ________________________ Signature ________________

Name of Health Facility____________________

**Part I: Socio demographic characteristics**

| **Sr.No** | **Question** | **Code ---------** | **Remark** |  |
| --- | --- | --- | --- | --- |
| 1 | Age | --------------years |  |  |
| 2 | Residence of respondent | 01. Urban 02.Rural |  |  |
| 3 | Marital status | 01. Single 02. Married 03. Divorced 04. Widowed  05. Separated | If not married skip Q11and 12 |  |
| 4 | Religion | 01. Protestant 02. Muslim 03. Orthodox 97. Others(specify) ----------- |  |  |
| 5 | Ethnicity | 01. Oromo  02. Amhara 03. Tigre  04. Gurage  97. Others(specify) ------------- |  |  |
| 6 | Occupation of the mother | 01. House wife 02. Farmer 03. Daily laborer 04. merchant  05. Government employee  06. private employee  07. Student 97. Others(specify) ------------- |  |  |
| 7 | Educational status of the mother | 01.Can’t read and write  02.can read and write 03.primary school (1-8)  04.secondary school(9-12)  05.college and above |  |  |
| 8 | Monthly house hold income | ------------ET Birr |  |  |
| 9 | How far your home from this institution? | -------------hrs. |  |  |
| 10 | Family Size | ___________ |  |  |
| 11 | Husband’s occupation | 01. Farmer 02. Daily laborer 03. Government employee  04. private employee  05.merchant  06. Student 97. Others(specify) ------------- |  |  |
| 12 | Husband’s educational status | 01.can’t read and write  02.can read and write  03.primary school (1-8)  04.secondary school(9-12)  05.college and above |  |  |

**Part II: Past obstetrics related information**

| 13 | Is it your first time pregnancy? | 01. Yes 02. No | If yes Go to  Qno.20 |
| --- | --- | --- | --- |
| 14 | How many times you became pregnant? | __________ |  |
| 15 | How many times you gave birth? | __________ |  |
| 16 | Is any of your pregnancies resulted in a baby that was born dead? | 01. Yes 02. No |  |
| 17 | Is any of your previous pregnancy ended with abortion? | 01. Yes 02. No |  |
| 18 | Have you had antenatal care for the past pregnancies? | 01. Yes 02. No |  |
| 19 | Was there any complication in previous pregnancy/ies, labor and delivery/ies? | 01.Yes 02.No |  |

**Current pregnancy related questions**

| 20 | By what means you recognized that you are pregnant? | 01.Missing period 02.Urine test 97.Others(specify)______ |  |
| --- | --- | --- | --- |
| 21 | Is this pregnancy approved by your husband and family? | 01. Yes 02. No |  |
| 22 | Is this pregnancy planned? | 01. Yes 02. No |  |

**Part III: ANC visit time and individual related information**

| 23 | For this pregnancy, at what gestational age you first booked to antenatal care? | ___________ Months |  |
| --- | --- | --- | --- |
| 24 | Why you booked during this/that period? | 01. I thought it is the right time 02. Misunderstanding of the right time and its purpose 03. I didn’t know that I’m pregnant 04. Ignorance 05. Others (specify) __________ |  |
| 25 | Have heard existence of ANC service? | 01..Yes 02..No |  |
| 26 | Did you get any information when to book to first ANC? | 01. Yes 02. No |  |
| 27 | When do you think the appropriate time for first antenatal care booking? | At ___________ Months |  |
| 28 | Who are your sources of information? | 01. Health workers  02. friends/relatives  03. Mass media  04. others (specify)------------- |  |
| 29 | Are you a member of WHDA (1-5network)? | 01. Yes  02. No |  |
| 30 | Is there ambulance service in your area? | 01. Yes  02. No |  |

**Questions used to asses’ decision making status of women**

| **S. no** | **Questions** | **Code** | **Remark** |
| --- | --- | --- | --- |
| 31 | Who is in your family usually has final say in the following decisions  1. Your own health care?  2. Using Current ANC | 01 02 03 97  Women alone Husband Jointly Others  -------- ---------- -------- ------  -------- ---------- -------- ------ |  |

**Thank you very much!!!**
